# Learning to Read in an Intermediate Depth Orthography: The Longitudinal Role of Grapheme Sounding on Different Types of Reading Fluency

**DOI:** 10.3390/bs14050396

**Published:** 2024-05-10

**Authors:** Sandra Fernandes, Luís Querido, Arlette Verhaeghe

**Affiliations:** 1 Faculdade de Psicologia, Universidade de Lisboa, Alameda da Universidade, 1649-013 Lisboa, Portugal; averhaeghe@psicologia.ulisboa.pt; 2 CICPSI, Faculdade de Psicologia, Universidade de Lisboa, Alameda da Universidade, 1649-013 Lisboa, Portugal; 3 Egas Moniz Center for Interdisciplinary Research (CiiEM), Egas Moniz School of Health & Science, Caparica, 2829-511 Almada, Portugal; lquerido@egasmoniz.edu.pt

**Keywords:** phonemic awareness, grapheme sounding, decoding fluency, word list fluency, text fluency

## Abstract

Phonological processing skills, such as phonological awareness, are known predictors of reading acquisition in alphabetic languages with varying degrees of orthographic complexity. However, the role of multi-letter-sound knowledge, an important foundation for early reading development, in supporting reading fluency development remains to be determined. This study examined whether two core foundational skills, phonemic awareness and grapheme sounding, have a predictive role in reading fluency development in an intermediate-depth orthography. The participants were 62 children learning to read in European Portuguese, and they were longitudinally assessed on phonemic awareness, complex grapheme sounding, and reading fluency (decoding, word, and text) from Grade 2 to Grade 3. The results showed that grapheme sounding predicted reading fluency development controlled for nonverbal intelligence and vocabulary, short-term verbal memory, and phonemic awareness. Grapheme sounding plays a prominent role in predicting reading fluency outcomes, whereas phonemic awareness (both accuracy and time per correct item) did not contribute to any of the three types of reading fluency. The fact that grapheme-sounding predicted reading fluency is likely due to complex grapheme-phoneme correspondences being required to achieve proficient reading. These findings provide insights into the cognitive processes underlying reading development in intermediate-depth orthographies and have implications for early literacy instruction.

## 1. Introduction

Phonological processing, that is, the use of phonological information in processing written and oral language, plays a crucial role in reading development [1]. Phonological awareness, phonological short-term memory, and rapid automatized naming (RAN) are three different components of phonological processing that can predict reading acquisition rates in several alphabetic languages with varying degrees of orthographic complexity (e.g., [1,2,3,4,5]).

Research has consistently shown a close association between phonological awareness and RAN and children’s reading acquisition with a longitudinal prediction of reading skills (e.g., [6], in five alphabetic orthographies with varying degrees of consistency), with short-term verbal memory having less or no predictive importance (e.g., [7]). Then, phonological awareness and RAN explain unique variances in children’s reading skills beyond general factors such as age and nonverbal IQ (e.g., [8]).

Phonological awareness, the conscious awareness of the linguistic units (e.g., rhyme, syllable, and phoneme) of speech, plays a crucial role in early literacy development in all alphabetic writing systems (e.g., [1]). Phonological awareness and specifically phonemic awareness (PA), which encompasses skills like isolating, segmenting, blending, and manipulating phonemes, the smallest units of spoken language, is thought to aid the child in connecting spoken sounds to written letters/graphemes and letter combinations. This connection forms the foundation for word reading and spelling proficiency. In inconsistent orthographies, competent translation between graphemes and phonemes is typically achieved earlier, and literacy skills grow faster than in inconsistent orthographies such as English (e.g., [9,10]). Consequently, the relatively limited impact of phonological awareness on reading proficiency in consistent orthographies may be attributed to the more efficient and earlier acquisition of consistent grapheme-phoneme correspondences in these languages. In European Portuguese (EP), an orthography with an intermediate level of orthographic consistency, Reis et al. [11] found that phonological awareness is the most important predictor of reading accuracy and fluency in the early beginning of literacy acquisition, but its weight decreased as schooling increased (beyond Grade 3). They also showed that, simultaneously, there was an increase in the contribution of variables associated with automatism and lexical recognition (i.e., RAN and Vocabulary). According to the authors, these results suggest that throughout learning, there is a dynamic alteration of the cognitive processes underlying reading, in which the child goes from a reading based on sub-lexical processes to a reading massively resorting to the orthographic recognition of words. In their concurrent study of several orthographies (Hungarian, Dutch, and Portuguese), Vaessen et al. [12] also stated that the extent to which phonological awareness and RAN contribute to word reading fluency varies depending on the level of reading expertise. Thus, in the case of EP, it was found that RAN plays a more significant role in the later stages of reading proficiency (beyond Grade 3). However, in the literature, some researchers argued that RAN is more important in the first two grades (e.g., [13]; see also [8]), while others claimed that RAN continues to predict reading even in higher grades, growing its impact over time (e.g., [14]). Additionally, there have been mixed findings regarding the growth patterns in reading that RAN contributes to [15].

Several studies have revealed that phonological awareness and RAN are linked to specific aspects of literacy processing, in addition to having presumably different weights throughout development and as a function of orthographic consistency. Specifically, phonological awareness is strongly associated with decoding skills (reading accuracy), whereas RAN is closely connected to reading fluency (e.g., [16,17]).

Beyond these two foundational skills, it has been recognized that letter-sound knowledge is also a core foundation (e.g., [18,19], a meta-analytic review [20]) that contributes to a child’s early literacy (e.g., [21]). Recently, Clayton et al. [22] showed that letter-sound knowledge was an independent, strong predictor of early reading development, along with phoneme awareness and alphanumeric RAN. Indeed, letter-sound knowledge underlies the ability to use the alphabetic principle, which is fundamental for decoding (e.g., [23]), whether inconsistent or inconsistent orthographies (e.g., [24]). Letter-sound knowledge is recognized to enable children to phonologically decode written words that are unfamiliar (e.g., [18]). Learning to read in the alphabetic system begins with this knowledge of grapheme-phoneme correspondences, which presupposes identifying letters and analyzing speech as a sequence of phonemes (e.g., [25]). This is patent in pseudoword reading when the child needs to identify the letters, group them into graphemes (e.g., “*tilho*” is divided into “*t-i-lh-o*”), match each grapheme with a phoneme, and finally correctly pronounce the pseudoword. The alphabetic principle and early word reading skills are more challenging to learn in inconsistent orthographies than in languages with more regular orthographies (e.g., [26]). It is well known that shallow orthographies, with one-to-one grapheme-phoneme correspondences, make decoding easier to learn (e.g., [10]).

In the continuum of orthographic depth, the EP orthographic code is more transparent than the French one, although more similar to the French than to the English [27,28,29,30]. As referred by Fernandes et al. [31]: “For example: (i) French has a much greater number of digraphs or complex graphemes representing a single phoneme and not a diphthong (pp, bb, tt, th, dd, dh, cc, ck, ch, gg, mm, nn, gn, gu, ng, ff, ph, ss, sc, rr, ll, ou, eu; a, i, o, u + n or m) than EP (nh, lh, ch, rr, ss, gu, qu; a, e, i, o, u + n or m); (ii) French allows many ways for spelling vocalic digraphs (e.g.,: nasal vowels: 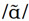 may be written an, am, en, em, …; and /$\tilde{\varepsilon }$/ may correspond to in, im, en, ein, ain, …) whereas EP allows a maximum of three alternate ways for spelling these same vocalic digraphs (e.g.,: nasal vowels: e, i, o, u + n or m, and a+n or m or ∼)” (p. 5).

EP orthography shows more significant inconsistency in phoneme–grapheme conversion (involved in the spelling process) than in the reverse conversion (involved in the reading process). In addition, the EP writing system uses diacritics to enhance the transparency of spelling–sound relations [32].

The grapheme-phoneme correspondences (GPCs) indeed depend on the complexity of the grapheme, with single letter graphemes (e.g., t, p) being distinct of multiple letters graphemes (e.g., nh, lh) or letters with diacritics (e.g., ê, ã). Single-letter graphemes are individual letters that represent distinct phonemes or sounds in a language without any additional marks. Letters with diacritics are letters that have additional marks attached to them, such as accents (e.g., é) or tilde (e.g., ã). Single-letter GPCs are straightforward because one letter maps onto a phoneme, while multiple-letter GPCs are complex as they have at least two letters that map onto a phoneme. Letters GPC with diacritics are also complex since each diacritic modifies the pronunciation of the base letter in a specific way. Learning to read is influenced by these relationships. The EP writing system is of intermediate complexity (e.g., [31,33]), and children need to learn many other spelling units than the 26 single letters of the alphabet to pair to their respective sounds or phonemes. The single-letter GPC may support the early reading acquisition, while the proficient reading required to achieve reading fluency and reading comprehension of complex material can be based on multi-letter GPC (e.g., [34]). According to Larsen et al. [35], this assumption is reinforced by educational policies and instruction programs, which induce an order of teaching, first privileging the teaching of single-letter GPC before multiple-letter GPC. Consequently, this order delays the attainment of proficient reading, preventing the reading of words with complex graphemes. In fact, Fernandes et al. [31], in EP, observed an effect of complexity in reading and spelling (with an advantage for items that contained only simple graphemes) until the end of Grade 1. According to the results of this study [31], Portuguese children at the end of Grade 1 still resort to single letter-sound correspondences and experience difficulty in mastering more complex relations between letters and phonemes (i.e., with complex graphemes—more than one letter corresponding to one phoneme, in that study). Therefore, it is important to highlight the contribution of children’s other knowledge, namely complex graphemes, rather than single-letter knowledge, to reading acquisition. However, most studies have focused on single-letter sound knowledge as a predictor of reading acquisition, and fewer have examined the role of multiple-letter sound knowledge.

Efficient word recognition then involves three processes that, though logically successive, are to some extent overlapping (e.g., [31]): (1) the acquisition of a (sequential and controlled) decoding procedure, which takes the orthographic rules of the particular language into account and proceeds from the smallest units to syllables, and to syllabic and non-syllabic phonograms; (2) the acquisition of stored orthographic representations of known words, which are activated automatically from the graphic input and lead, also automatically, to the activation of the corresponding semantic referents and syntactic functions; and (3) the acquisition of the ability to match text-structure cues, including punctuation, to stored knowledge of grammar and oral language prosody. Testing this knowledge is necessary to determine whether reading difficulties stem from a poor grasp of graphemes. Some authors (e.g., [36,37]) believe that a failure to automatize associations between speech sounds and letters is a proximal cause of difficulties in learning to read. However, for the learner to be able to decode words, fluency in producing sounds that correspond to graphemes is not enough. Sounds associated with successive letters in an acceptable spelling pattern must be represented at an abstract, phonemic level to be blended and thus yield the pronunciation of a written word or potential word. Fluency of decoding increases when the reader elaborates phonological representations that can be accessed directly, that is, without the sequential blending of graphemes. These representations correspond to complex onsets or codas, rimes, syllables, and more generally to phonograms, that is, letter groups within a word that share the same pattern across words.

Oral reading fluency is essential to reading mastery (e.g., [38,39,40]). Although usually defined as text-reading efficiency or automaticity (e.g., [38,41,42]), measuring oral reading fluency involves evaluating a person’s accuracy and rate when reading at the sub-lexical, lexical, and discourse levels. Thus, three types of reading fluency can be distinguished, depending on the material used: (1) lists of pseudowords, (2) lists of unrelated words, and (3) connected meaningful text presented as either a list of single sentences or an organized set of related sentences (e.g., [42,43]). Whichever the material, oral reading fluency is a measure of speed under the condition of accuracy, often expressed as the number of correct words or pseudowords read aloud in one minute (e.g., [39,44]).

The three types of reading fluency mentioned involve differentiated constituent skills; indeed, one does not read one or more sentences as one reads a list of words or a list of words as a list of pseudowords. Parallel processes at sub-lexical, lexical, and textual levels support text reading fluency (see, e.g., [45]). Whereas sub-lexical and lexical processes are involved when reading unrelated words, only sub-lexical processes, usually called decoding, intervene in pseudoword reading (e.g., [46]). We must use pseudowords—letter sequences consistent with the particular language’s phonotactics, which the reader supposedly never met before—to ensure that the reading mechanism uses graphophonological correspondences (i.e., that there occurs no access to lexical memory). The three measures of reading fluency (pseudoword, word, and text) can be used to assess reading ability from Grade 1 to subsequent school years. However, the relative contributions of the emergent reading skills to the three types of fluency change depending on the level of reading development. Thus, understanding changes in reading fluency requires understanding how reading ability develops. There are two reasons why reading lists of words may be faster and more accurate than reading lists of pseudowords: in beginning readers, the decoding may be supplemented or corrected by knowledge of the spoken word (e.g., when there is no rule that indicates a given word’s correct pronunciation, the child may read, for example, “*máximo*” [maximum] and “*táxi*” [taxi], because, being familiar with the words, s/he infers that the “x” must be read/s/and/ks/, respectively); in more advanced readers, words may be recognized automatically through the activation of stored representations in the mental orthographic lexicon (e.g., [34,42]).

Text reading fluency is expected to be the highest, particularly in more skilled readers. When reading fluency is calculated using a text rather than a word list composed of that same text’s words, text fluency will be enhanced by the expectations engendered by syntactic and semantic sentential constraints. Even if the task does not require explicit text comprehension, when the task is one of merely reading a text aloud, grammaticality, lexical restrictions, and local semantic processing may provide sufficient cues for the participant to read the same words faster in a text than in list form. Jenkins et al. [42] found that reading words in context (without specific cuing for comprehension) explained much more variance in reading comprehension than reading the same words in a list (70% vs. 9%) (see also, [33]).

Decoding is crucial for the development of word reading, and conscious knowledge of the smallest units (phonemes and graphemes) that encode speech and letter-sound knowledge is necessary for accurate decoding (e.g., [46]). In addition, learners need to have a robust letter-sound knowledge base in order to grasp complex spellings such as consonant blends [21].

In the present study, we aimed to test whether two core foundational skills, PA and Grapheme Sounding (GS), have a predictive role in reading fluency. Based on previous research (e.g., [11,24]), we hypothesize (H1) that both PA and GS will be significant predictors of reading fluency development, with GS making an independent contribution beyond that of PA. It is also hypothesized (H2) that the contribution of time per correct item measures of GS and PA will be higher than the accuracy ones (e.g., [36]). Considering that the outcome measures are collected in an intermediate level (Grade 3) of reading acquisition, we finally hypothesize (H3) that the pattern of effects would change as a function of the type of reading fluency (e.g., [45]), i.e., higher predictive effects of PA and GS, on word reading fluency and text reading fluency than on decoding fluency.

By investigating these hypotheses, we hope to provide insights into the underlying mechanisms of reading acquisition and development in EP.

These relations were tested with separate hierarchical regression analyses (HRAs) for longitudinal relationships between two test periods, one at each grade level.

We used a test of vocabulary knowledge and a test of immediate verbal memory as controls for language ability. However, vocabulary knowledge is considered to facilitate written word recognition [11,33,47] as well as to be improved by the latter [48,49]. Likewise, immediate verbal memory is considered to support reading [50,51] and to be influenced by reading [52,53,54]. For these reasons, we also looked for the potential influences of those two reading-instrumental language variables on oral reading fluency.

The present study was carried out in an intermediate-depth orthography, which adds to the literature in several important ways. Two critical emergent literacy skills—PA and GS—were analyzed as predictors of oral reading fluency. To become proficient readers, children must learn both single- and multiple-letter GPC. However, most of the studies to date have only focused on children’s single-letter knowledge. The task used for assessing GS has been designed specifically for this project since there is none of this nature in EP. Moving forward, this task will be available to integrate assessment batteries for emergent reading skills. The present study aims to be the first in EP to explore the children’s knowledge of a complex set of graphemes (e.g., multiple-letter graphemes, letters with diacritics). Literacy instruction will, therefore, benefit from it. Furthermore, to our knowledge, there have been no longitudinal studies on these relationships in EP.

## 2. Materials and Methods

### 2.1. Participants

Data reported in the present study come from a larger project on reading and spelling development in EP. The Ministry of Education of the Portuguese Government made the schools that participated in this project available.

A total of 62 children (of whom 30 were girls) from two public schools in the Lisbon district participated in this study. Following ethical APA guidelines, parents of all participating children provided informed consent consistent with the Office of Statistics and Planning of Education of the Ministry of Education. The cohort was tested in three testing periods, more precisely throughout the initial three and the final three months of the school year in Grade 2 and in the last three months of Grade 3. Children’s mean age at the end of Grade 2 was 96.45 (SD = 3.30) months. Students came from two different classrooms randomly chosen per Grade in each school. They were all native speakers of EP and had average or above average cognitive functioning—25th percentile or higher on the Raven’s Coloured Progressive Matrices [55]. Children flagged by their parents and teachers as having learning, emotional, or sensory disabilities were not included in the sample. This study’s participants comprise children from families with a middle to upper-middle socioeconomic status, as determined by a questionnaire that was completed by the parents of the children. Teachers employed a phonics-based approach for reading instruction.

### 2.2. Measures

#### 2.2.1. Verbal and Nonverbal Abilities

Vocabulary Peabody Picture Test (VPPT). VPPT was adapted from the Spanish version [56]. In each item, four images with different meanings are presented, and the participant must point to the picture that corresponds to a given spoken word. According to Dunn et al. ([56], p. 90), concurrent validity with other vocabulary measures (e.g., Standford-Binet Vocabulary Subtest) was 0.71. Test–retest reliability over a 1-and-a-half-year interval reported by Fernandes et al. [33] was 0.50.

Raven’s Colored Progressive Matrices (RCPM). The general nonverbal cognitive ability was assessed using RCPM Portuguese adaptation [55]. The reported Cronbach alpha was 0.91.

#### 2.2.2. Oral Sentence Immediate Recall

There is evidence of links between phonological short-term memory and immediate recall of sentences (e.g., [50,57,58,59,60]). In the absence of a phonological short-term memory task in the extended project, we used a sentence immediate recall task for the present study.

A set of 13 sentences was given to each child, each with a different grammatical structure. (e.g., “*O cão velho de orelhas grandes que está encostado ao cachorro tem coleira*”. [The old dog with large ears that is leaning against the puppy has a leash], and “*O avião com janelas pequenas tem hélices, mas não está a levantar voo*”. [The plane with small windows has propellers but is not taking off.]. All sentences were 13 words long. The experimenter presented each sentence, one by one, through an mp3 player and Creative speakers. Children were asked to recall each sentence immediately, word by word. Responses were tape-recorded, and subsequent scoring was based on these recordings. A word in a sentence was only scored as correct if it was recalled in its original position within the sentence and pronounced correctly. The test–retest reliability score over a one-year interval was 0.79.

#### 2.2.3. Phonemic Awareness (PA)

Phoneme Deletion Test. Children were asked to say aloud the phonological segment obtained after deleting the initial phoneme from a consonant cluster of a monosyllabic pseudoword. Ten orally presented CCV stimuli (e.g., *blu* and *fla*) were presented over headphones. Experimental items were preceded by three familiarization trials, which could be re-administered if the child failed to understand the task or asked the experimenter to repeat any part of the instruction. Stimuli presentation was controlled by E-Prime 1.1 [61,62] running on a Pentium PC. The reliability of the test [63] was 0.87 (Cronbach alpha).

#### 2.2.4. Grapheme Sounding (GS)

The child was asked to read aloud (“say the sound”)—in an efficient and speedy way—complex graphemes consisting of one letter with diacritic (á, é, ó, ã, ê, ô, ç), two letters with diacritics (oral and nasal diphthongs: éu, ói, ão, ãe, õe, ân, âm, ên, êm) and without diacritics (ss, rr, nh, lh, ch, plus oral and nasal diphthongs: ai, ei, oi, ui, au, eu, ou, an, am, in, im, un, um, en, em, on, om) presented in lowercase and randomly displayed in columns. The accuracy (proportion of correct items) and the time per correct item were used for analysis. The test demonstrated an acceptable level of reliability, as indicated by Cronbach’s alpha values, which stood at 0.63.

#### 2.2.5. Reading Fluency

Text reading fluency. The text was written in a Times New Roman 16 font type with “normal” spacing between letters and 1.5 spacing between lines. The text was selected from school manuals and was not used as textbooks in the participating schools, but was appropriate. The readability level of the text was evaluated with the Flesch Reading Ease Formula adapted for the Spanish language. In the absence of any readability formula for Portuguese, we adopted the Spanish adaptation of Flesch Reading Ease Formula [64]. The European Portuguese language has a greater level of similarity concerning the frequency of mono and multisyllabic words with Spanish than with English or French. Test–retest reliability over a 1-year period reported by Fernandes et al. [33] was 0.88. To assess the two dimensions of reading fluency (accuracy and automaticity), the children were asked to read aloud in an efficient and speedy way to comprehend “what the text said”. Errors and reading times were recorded. A digital mp3 recorder was utilized to obtain oral reading recordings. The number of correct words per minute (rate) was used for analysis.

Word reading fluency. The words were the same as in the text presented for text reading fluency but presented in columns in a pseudo-randomized order to prevent the appearance of contextual and/or semantic relationships between the words. The child was asked to read the words as rapidly and efficiently as possible. The number of words read correctly and the reading times were recorded in a way similar to that used for text reading fluency. Test–retest reliability over a 1-year period was 0.83.

Decoding/pseudoword reading fluency. Pseudowords were constructed from the words of the word reading fluency test so that both tests had the same number of items. The order of the pseudowords was randomized. The presentation and procedure were similar to those used for the word reading fluency test. Test–retest reliability over a 1-year period was 0.82.

### 2.3. General Procedure

The tests were conducted by a team of trained graduate students in a quiet environment provided by the school. The Raven’s Colored Progressive Matrices (RCPM) test was administered to groups of participants. The Vocabulary Peabody Picture test, PA, GS, and oral sentence immediate recall were individually administered in random order.

For the RCPM and Vocabulary Peabody Picture tests, the participants were assessed at the beginning of Grade 2. The assessment of PA, GS, and Oral Sentence Immediate Recall was performed at the end of Grade 2.

All of the fluency measures were individually administered in different sessions in a fixed order (text, word, and pseudoword) and with at least a 1-week interval between the sessions. These assessments were performed during a several-week period at the end of Grade 3.

The students’ responses were recorded using a Memup mp3 recorder and played back later for analysis purposes (with the exception of the Vocabulary Peabody Picture Test, for which the students’ responses were registered on paper by the experimenter). The testers timed the students’ performance using digital count-down stopwatches.

## 3. Results

### 3.1. Descriptive Statistics and Correlational Analyses

Table 1 shows the mean and standard deviations for all measures for all testing periods.

A summary of concurrent and longitudinal correlations between measures is presented in Table 2 below.

Table 2 shows the correlation coefficients among all variables in Grade 2 and Grade 3. It is worth noting that Vocabulary has a significant correlation with words (*r* = 0.31) and text reading fluency (*r* = 0.29), and PA accuracy has a significant correlation with pseudoword (*r* = 0.29) and text reading fluency (*r* = 0.34). PA (time per correct item) correlated significantly with the word (*r* = 0.28) and text reading fluency (*r* = 0.29), whereas GS (time per correct item) correlated significantly with all reading fluency measures (*r* = 0.44, *r* = 0.53, and *r* = 0.43, respectively for pseudoword, word, and text reading fluency).

### 3.2. Hierarchical Regression Analyses (HRA)

Hierarchical regression analyses were subsequently conducted between the two grade levels to examine the contribution of PA and GS (accuracy or time per correct item measures, assessed in Grade 2) to reading fluency outcomes (pseudoword, word, and text reading fluency, assessed in Grade 3). In each regression model, non-verbal reasoning (RCPM) and vocabulary were entered first as control variables. The control variables introduced in the first step were not assessed at the same time as the independent variables. However, given their relatively stable nature over time, we chose to retain them as controls in the regression model, as they contribute to the robustness of the analysis. The sentence recall entered the regression equation at step 2 to control for contributions from memory abilities. PA entered the regression equation in step 3 and GS in step 4. The third step allowed us to control the unique contribution of GS beyond PA.

Table 3 and Table 4 show the standardized beta coefficients, *R*^2^ changes, and significance levels of the longitudinal regression analyses for accuracy and time per correct item measures of PA and GS predicting literacy measures.

For accuracy measures, after controlling for nonverbal intelligence and vocabulary, sentence recall, and PA, GS at the end of Grade 2 accounted for unique variance in decoding fluency (8%, *β* = −0.312, *p* < 0.05), in word fluency (8%, *β* = −0.321, *p* < 0.05) and in text fluency (6%, *β* = −0.276, *p* < 0.05) at the end of Grade 3. Sentence recall accounted for unique variance in text fluency (7%, but without a significant effect *β* = −0.135, *p* > 0.10) but not in decoding and word fluency. There were no significant contributions from PA for each reading fluency measure.

For time per correct item measures, after controlling for nonverbal intelligence and vocabulary, sentence recall, and PA, GS at the end of Grade 2 also accounted for unique variance in decoding fluency (16%, *β* = −0.409, *p* < 0.01), in word fluency (24%, *β* = −0.498, *p* < 0.001) and in text fluency (14%, *β* = −0.385, *p* < 0.01) at the end of Grade 3. Sentence recall (7%, *β* = −0.296, *p* < 0.01) accounted for a unique variance in text fluency, and PA (7%, but without a significant effect *β* = −0.205, *p* > 0.10) accounted for a unique variance in text fluency and word fluency. The last relationship must be taken into account with caution because the changes in R-squared might not be meaningful because the predictor PA does not provide a statistically significant contribution to the dependent variables—word and text reading fluency.

Concerning non-verbal reasoning (RCPM) and vocabulary, only the last accounted for unique variance in word fluency (*β* = 0.385, *p* < 0.01 and *β* = 0.256, *p* < 0.05, respectively, for accuracy and time per correct item analyses).

These results suggest that GS accounted for unique variance in all literacy measures, with the largest effects occurring from time per correct item measure.

## 4. Discussion

As research continues to expand in its discovery of the universal predictors of reading, this study sought to further investigate the importance of GS for reading fluency in relation to the key literacy universal phonological awareness. It is acknowledged that reading fluency, reflecting automaticity, plays a fundamental role in proficient reading with comprehension [33]. Many studies conducted early in literacy development demonstrate the predictive value of these two key skills: letter knowledge and PA. However, assessing letter knowledge, in a general sense, typically involves single letters, both named and/or sounded. Nevertheless, in most alphabetic writing systems, letter knowledge extends beyond single letters. Hence, there is a crucial need to assess grapheme sounding with complex multi-letter graphemes, especially in the advanced stages of learning to read. The present study sought to address this issue by assessing children at two distinct time points—during Grades 2 and 3 of their reading acquisition in EP, an intermediate-depth orthography. Thus, the primary aim of this study was to examine the longitudinal contribution of GS alongside that of PA for three types of reading fluency: decoding, word, and text.

Regarding the first hypothesis of this study, we anticipated that both PA and GS would predict the development of reading fluency, with GS making an independent contribution beyond that of PA. The results showed that GS, both with accuracy and time per correct item measures at the end of Grade 2, was a unique predictor of reading fluency outcomes (decoding, word, and text fluency) at the end of Grade 3 after controlling for non-verbal reasoning, vocabulary, and immediate verbal memory. On the contrary, PA (both accuracy and time per correct item) did not contribute to any of the three types of reading fluency, which does not fully support our first hypothesis.

The results revealing a unique contribution of GS in reading fluency outcomes were unsurprising, given that the literature pointed to the variability in the contribution of different phonological knowledge (e.g., multi-letter GPC) throughout reading development. Previous studies have already recognized letter-sound knowledge as a core foundation for early reading development [18,19,20]. In fact, letter-sound knowledge underlies the ability to use the alphabetic principle, which is fundamental for decoding [23].

The fact that GS predicted reading fluency in the present study is likely due to multi-letter GPC being required to achieve the proficient reading of complex material typical of a Grade level that is no longer the initial one (e.g., [34]). The previous literature (e.g., [12]) highlighted the complexity of the relationship between different emergent reading skills and reading fluency in orthographies varying the degree of opacity. Studies in orthographies with transparent languages (e.g., Finnish, Spanish) have found that letter-sound knowledge has an important predictive power for learning to read compared to phonological awareness (e.g., [65,66]). The present results reinforce this aspect of complexity, indicating that GS plays a more prominent role in the later stages of reading proficiency. In contrast, the absence of PA’s contribution to any reading fluency measure was somehow unexpected. However, Reis et al. [11] have already found that, in EP, the contribution of PA (accuracy) to reading decreased as schooling increased while the contribution of variables associated with automatism and lexical recognition increased. The GS adds to this set of variables that play a role in later stages of reading acquisition, particularly in reading fluency outcomes. In children learning to read in EP, a language with an intermediate depth orthography, decoding begins to be efficient in Grade 3 (see, for example, [33]). Regardless of orthographic depth, the explicit letter-sound knowledge of how the language sounds map onto the letters/graphemes, if fluent, facilitates automatic word recognition (e.g., [25,34]). We can presumably assume some automaticity in word recognition in third graders. This aspect may explain the contribution of GS, based on multi-letter GPC, to fluency measures that require reading proficiency.

We have also anticipated (H2) that the contribution of time for correct item measures of GS and PA will be higher than accuracy ones. The inclusion of a speed measure is of interest because it is a more discriminative tool that allows for further differentiation when accuracy ceiling levels are attained (e.g., [36,67]), i.e., in more advanced grades. As children progress in their reading development, they develop automaticity in phonological processing (e.g., [34]). Speed measures capture the efficiency and automatic nature of these processes, reflecting the ability to manipulate and process phonological information quickly. They could reveal variations in how quickly readers process phonological information, providing a more nuanced understanding of their phonological processing abilities. Adding speed measures to the accuracy ones can offer a more comprehensive picture of phonological processing skills in different stages of reading development.

Although PA did not present any influence on reading fluency, the unique variance explained by GS time per correct item measure was particularly prominent and higher than that observed with the accuracy measure, emphasizing its significance in the development of efficient reading. These effects were consistent even after accounting for nonverbal intelligence, vocabulary, sentence recall, and PA. Thus, the assumption that the contribution of time for correct item measures of GS and PA will be higher than accuracy ones was partially supported.

The third hypothesis of this study aimed to examine whether the pattern of effects would change as a function of the type of reading fluency, i.e., higher predictive effects of PA and GS on word reading fluency and text reading fluency than on decoding fluency. The analysis of the results revealed that there were no noticeable changes in the influence pattern of either PA or GS, as measured by accuracy and time per correct item, as a function of the type of reading fluency. These results are not in line with the expected (H3). The contribution of PA was non-existent, as already referred, and the contribution of GS was roughly the same for the three reading fluency outcomes. Contrary to what happens with PA, whose effect decreases with schooling, GS appears to be a predictor that exerts its influence until later stages and equally across various reading processing mechanisms, including decoding, word recognition, and meaningful, connected text reading. While the results may not have aligned with our initial expectations, they are still significant in providing valuable insights for our future research on exploring the impact of GS on reading development.

Short-term verbal memory, considered a component of phonological processing, has been identified as a weak predictor of reading acquisition (e.g., [7]). In our study, phonological short-term memory, assessed through immediate sentence recall, demonstrated a significant contribution to one of the fluency measures—text reading fluency. This suggests a potential impact of memory abilities on the ability to read connected and meaningful text fluently. According to Alloway and Gathercole [50], sentence recall is supported both by conceptual representations and by the short-term memory processes. During a sentence recall task, phonological, lexical, and semantic levels of representation are activated (e.g., [57]). Baddeley [68] proposes that sentence memory is handled by the episodic buffer in working memory. This system integrates inputs from various working memory components and other cognitive systems, representing inputs in a multi-dimensional code. For sentences, it combines the phonological representation with conceptual representations derived from language processing [50]. The influence identified in our study regarding sentence recall on text-reading fluency may be due to the close connection between phonological processing and the decoding and pronunciation skills necessary for fluent text reading. Additionally, the importance of working memory is highlighted, as it is crucial for processing and comprehending longer passages of connected text, ultimately contributing to text reading fluency. Lastly, the alignment of the ability to retain meaning and comprehend with the higher-order skills involved in text reading fluency could also contribute to this influence.

Overall, the results obtained provide an insightful analysis of the unique contribution of GS to different measures of reading fluency, aligning with the outlined objectives, hypotheses, and the reviewed scientific literature.

Some limitations of the present study should be acknowledged. First, despite the longitudinal design of the present study, significant relations between the measures do not imply causation. Second, as mentioned above, although the RAN, in previous studies in EP, has contributed to reading measures only from the 4th year onwards, to be not included in the present study, it does not allow us to make any comments concerning its potential contribution. Third, we have differences in timing measures between the GS and PA tasks, as observed in Table 1. These tasks employ different time measures; however, our focus lies not in directly comparing processing time but rather in investigating how the time associated with each task contributes to various measures of reading fluency. Fourth, we acknowledge limitations stemming from our sample size in computing Cronbach’s alpha coefficients, which could have been more accurately calculated with a larger sample. Nevertheless, the literature regarding sample size requirements for reliability studies lacks consensus, and recommendations for sample size vary widely, with some suggesting smaller samples may suffice while others advocate for larger samples. Furthermore, it is widely accepted that Cronbach’s alpha coefficients can be applied to cognitive performance measures with relatively smaller sample sizes compared to psychological self-report scales. Finally, our participants were only from a cohort of Grade 2 to Grade 3 and did not cover all the stages of learning to read.

Future research may explore the longitudinal relationships between these emergent literacy skills and later reading outcomes covering a wider range of grade levels. Additionally, extending the longitudinal analysis to include measures of reading comprehension would provide a more comprehensive understanding of the role (direct and/or mediated) of GS in long-term reading development. Furthermore, investigating instructional strategies that specifically target GS could provide valuable insights into effective approaches for fostering reading fluency in children learning to read, particularly in intermediate-depth orthographies, such as EP. Additionally, future research could explore the progression of either PA or GS in readers with learning difficulties, particularly those with dyslexia, compared to typical readers.

## 5. Conclusions

This study examined the longitudinal contributions of GS and PA to the different types of reading fluency in EP. GS emerged as the sole predictor, surpassing PA in both accuracy and time measures. The role of immediate sentence recall in text reading fluency further adds complexity to the intricate interplay of skills involved. These findings contribute to our understanding of the unique contribution of GS to different types of reading fluency, shedding light on the intricacies of early literacy development in the context of EP and providing valuable insights for educators, researchers, and professionals involved in promoting literacy.

## Figures and Tables

**Table 1:** Descriptive statistics for all variables across grades.

	Grade 2						Grade 3		
	Beginning (November–January)			End (April–June)			End (April–June)		
	Mean	SD	Range	Mean	SD	Range	Mean	SD	Range
RCPM	28.90	4.08	20–36						
Peabody Vocabulary	124.66	11.04	90–145						
Sentence Recall				0.72	0.11	0.31–0.92			
Grapheme Sounding (accuracy)				0.90	0.08	0.65–1			
Time per correct item ^a^				1.31	0.39	0.68–2.85			
Phoneme Deletion (accuracy)				0.85	0.19	0.20–1			
Time per correct item ^b^				3227.55	1317.82	1273–7068			
Text Reading Fluency (rate)							104.42	27.02	34.85–182.01
Word Reading Fluency (rate)							76.89	18.22	34.18–124.06
Pseudoword Reading Fluency (rate)							45.29	12.38	20.30–72.74

^a^ Time of correct sounding (in seconds); ^b^ Time lapsed between the stimulus presentation and the beginning of response (in milliseconds); Note: rate = number of correct items per minute.

**Table 2:** Correlation matrix for measures.

	Variable	1	2	3	4	5	6	7	8	9	10
Beginning of Grade 2											
1	RCPM	1									
2	Vocabulary	0.199	1								
End of Grade 2											
3	Sentence Recall	0.223	0.424 **	1							
4	Phonemic Awareness accuracy	0.131	0.187	0.387 **	1						
5	Grapheme Sounding accuracy	0.179	−0.243	0.156	0.020	1					
6	Phonemic Awareness time per correct item	−0.098	−0.079	−0.001	−0.210	−0.204	1				
7	Grapheme Sounding time per correct item	−0.031	0.001	−0.033	0.036	−0.650 **	0.174	1			
End of Grade 3											
8	Pseudoword Reading Fluency	0.048	0.172	0.205	0.285 *	0.244	−0.237	−0.441 **	1		
9	Word Reading Fluency	0.070	0.309 *	0.226	0.237	0.210	−0.280 *	−0.531 **	0.835 **	1	
10	Text Reading Fluency	0.204	0.288 *	0.382 **	0.342 **	0.250	−0.290 *	−0.432 **	0.776 **	0.768 **	1

* *p* < 0.05; ** *p* < 0.01.

**Table 3:** Coefficients of longitudinal hierarchical regression analyses (accuracy measures) predicting literacy measures.

		Decoding Fluency		Word Fluency		Text Fluency	
Step		*β*	Δ*R*^2^	*β*	Δ*R*^2^	*β*	Δ*R*^2^
1	RCPM Vocabulary	−0.082	0.03	−0.080	0.10 ^a^	0.045	0.11 *
		0.225		0.385 **		0.246	
2	Sentence recall	−0.020	0.02	−0.041	0.01	0.135	0.07 *
3	Phonemic Awareness	0.255	0.05	0.185	0.02	0.233	0.04
4	Grapheme Sounding	0.312 *	0.08 *	0.321 *	0.08 *	0.276 *	0.06 *
Total *R*^2^			0.18 *		0.22 *		0.28 **

* *p* < 0.05; ** *p* < 0.01; ^a^ *p* = 0.052.

**Table 4:** Coefficients of longitudinal hierarchical regression analyses (time per correct item measures) predicting literacy measures.

		Decoding Fluency		Word Fluency		Text Fluency	
Step		*β*	Δ*R*^2^	*β*	Δ*R*^2^	*β*	Δ*R*^2^
1	RCPM Vocabulary	−0.035	0.03	−0.039	0.10 ^a^	0.080	0.11 *
		0.101		0.256 *		0.131	
2	Sentence recall	0.156	0.02	0.110	0.01	0.296 *	0.07 *
3	Phonemic Awareness	−0.161	0.05	−0.177	0.07 *	−0.205	0.07 *
4	Grapheme Sounding	−0.409 **	0.16 **	−0.498 ***	0.24 ***	−0.385 **	0.14 **
Total *R*^2^			0.27 **		0.42 ***		0.39 ***

* *p* < 0.05; ** *p* < 0.01; *** *p* < 0.001; ^a^ *p* = 0.052.

## Data Availability

Data are contained within the article.
